# LEVERAGING CONSUMER HEALTH TECHNOLOGIES TO ENGAGE CARE PARTNERS OF OLDER ADULTS WITH DEMENTIA

**DOI:** 10.1093/geroni/igad104.3449

**Published:** 2023-12-21

**Authors:** Jamie Smith, Jessica Cassidy, Catherine Riffin, Jennifer Wolff, Hillary Lum

**Affiliations:** Johns Hopkins University, Baltimore, Maryland, United States; University of Texas at Arlington, Arlington, Texas, United States; Weill Cornell Medicine, New York City, New York, United States; Johns Hopkins University, Baltimore, Maryland, United States; Univerisity of Colorado School of Medicine, Aurora, Colorado, United States

## Abstract

Care partners are critical in supporting the complex health needs of older adults with dementia; the importance of their role increases with disease progression. Despite their extensive involvement in dementia care, care partners are not systematically identified or supported in healthcare settings. Consumer health information technology (CHIT) affords a largely untapped opportunity to formalize dementia care partner identification and engagement in healthcare delivery. One example is OurNotes, a portal-based agenda-setting questionnaire that allows patients to submit concerns for discussion ahead of medical visits. In a proof-of-concept study, OurNotes was first adapted to elicit the identity of the person completing the form and clarify their relationship to the patient, and subsequently piloted at an academic geriatric practice. Semi-structured interviews were conducted with 15 care partners to elicit their perceptions of the adapted OurNotes (utility, benefits, challenges) and suggestions for refinement. Transcripts were analyzed using deductive, thematic analysis. Care partners’ perceptions of the tool were overwhelmingly positive. Major benefits included enhancing their feelings of preparedness for the visit, having a voice to express concerns, and streamlining visit communication. The most commonly endorsed challenge was the time required to complete the adapted OurNotes in addition to routine check-in items. A primary suggestion was to enable the submitted OurNotes responses to be edited/updated. Findings suggest that the adapted OurNotes is an acceptable approach to identifying care partners in the dementia context. Refinements may further facilitate visit efficacy through preparedness. Future research will examine CHIT engagement on patient and care partner outcomes.

